# Musculoskeletal discomfort, urinary incontinence, and health-related quality of life in female farmers aged 40–65 years: a cross-sectional comparative study from rural Türkiye

**DOI:** 10.1186/s12905-026-04682-x

**Published:** 2026-07-17

**Authors:** Meşküre İçel, Doğukan Kurç, Ayşe Nur Tunalı, Seda Saka

**Affiliations:** 1 Department of Physiotherapy and Rehabilitation, Institute of Graduate Education, Haliç University, Istanbul, Türkiye; 2 Department of Physiotherapy and Rehabilitation, Faculty of Health Sciences, Istanbul Medipol University, Istanbul, Türkiye

**Keywords:** Female farmers, Musculoskeletal discomfort, Urinary incontinence, Quality of life, Occupational health, Rural health, Türkiye

## Abstract

**Background:**

Female farmers are exposed to combined biomechanical and pelvic-floor stressors during agricultural work. However, musculoskeletal discomfort, urinary incontinence, and health-related quality of life have seldom been evaluated together in this population. This exploratory study therefore compared these outcomes between female farmers and rural homemakers and examined associations between regional discomfort and quality of life.

**Methods:**

This cross-sectional comparative study included 70 women recruited by snowball sampling: 35 farmers and 35 homemakers aged 40–65 years from rural districts of Aydın Province, Türkiye. Outcomes were assessed using the Cornell Musculoskeletal Discomfort Questionnaire (CMDQ), the International Consultation on Incontinence Questionnaire-Short Form (ICIQ-SF), and the 36-item Short Form Health Survey (SF-36). Group comparisons, regression models adjusted for age, body mass index, and menopausal status, and Spearman correlations corrected for the false discovery rate (FDR; significance at q < 0.05) were performed.

**Results:**

Farmers reported a higher discomfort prevalence in 12 of 18 CMDQ regions. The largest unadjusted differences were observed for the left shoulder (48.6% vs. 26.5%; +22.1% points), left knee (65.7% vs. 45.7%; +20.0), hip (42.9% vs. 25.7%; +17.1), and right forearm (20.0% vs. 8.6%; +11.4). These differences were not statistically robust: no region remained significant after FDR correction (minimum q = 0.626), and adjusted logistic models showed no independent group associations (*p* = 0.081–0.993). ICIQ-SF scores tended to be higher among farmers (4.0 ± 3.8 vs. 3.0 ± 4.2; unadjusted *p* = 0.127; adjusted *p* = 0.578), and self-reported leakage during physical activity/exercise was more frequent (31.4% vs. 11.4%; *p* = 0.078). SF-36 domains were numerically lower among farmers, with no significant adjusted differences (adjusted p ranging from 0.296 for role emotional to 0.886 for physical functioning). FDR-significant inverse correlations included farmers’ upper-back discomfort with general health (rho=-0.700, q < 0.001), farmers’ right-knee discomfort with bodily pain (rho=-0.672, q < 0.001), and homemakers’ low-back discomfort with bodily pain (rho=-0.632, q = 0.003).

**Conclusions:**

In this exploratory rural sample, female farmers showed a broader distribution of musculoskeletal discomfort and a nonsignificant tendency toward more activity-related urinary leakage. Adjusted analyses did not confirm independent group effects; the results should therefore be interpreted as hypothesis-generating. They nonetheless suggest the value of considering combined musculoskeletal and urinary screening, and of more detailed exposure assessment, in rural women’s health.

**Supplementary Information:**

The online version contains supplementary material available at https://doi.org/10.1186/s12905-026-04682-x.

## Introduction

Agricultural work is physically demanding and has long been associated with a high burden of musculoskeletal disorders among farmers and agricultural workers [1]. Common agricultural tasks include lifting, carrying, repetitive upper-limb activity, kneeling, squatting, and prolonged standing. Working in such awkward and sustained postures may increase the mechanical load on the musculoskeletal system. These occupational exposures may be particularly relevant for women, who often combine agricultural labour with domestic responsibilities.

Systematic reviews and observational studies have reported a high prevalence of musculoskeletal symptoms among farmers, most commonly affecting the low back, knees, neck, shoulder, and upper extremities [2–5]. Heavy load carrying has additionally been linked to musculoskeletal pain and disability among rural women [6].

Urinary incontinence is another clinically relevant condition for adult women and may be influenced by age, body mass index, menopause, parity, and physical strain [7–11]. In rural agricultural contexts, repeated lifting and carrying, prolonged standing, squatting, and limited access to toilets may contribute to exertion-related or urgency-related leakage, while urinary symptoms may restrict work participation and quality of life. Conservative approaches, especially pelvic-floor muscle training, are therefore clinically relevant when urinary symptoms coexist with physically demanding work [8, 12–14].

Despite increasing evidence on occupational health in agricultural populations, few studies have simultaneously evaluated musculoskeletal discomfort, urinary incontinence, and health-related quality of life in female farmers, particularly in rural Turkish populations. The present study therefore compared women whose primary daily work was agricultural with homemakers from the same rural districts. We aimed to describe regional musculoskeletal discomfort, urinary incontinence, and quality-of-life patterns and to explore associations between body-region discomfort and quality-of-life domains. Given the cumulative physical and pelvic-floor demands of farming, we expected that farmers would report more widespread musculoskeletal discomfort and more frequent urinary leakage than homemakers; because this was an exploratory comparison in a small sample, these expectations were treated as hypotheses to be examined rather than as formally powered tests.

## Methods

### Study design and setting

This observational, analytical, cross-sectional comparative study was conducted between July and August 2021 in the Germencik and İncirliova districts of Aydın Province, Türkiye, including the villages of Ömerbeyli, Sınırteke, Erbeyli, and Arzular.

The study was performed in accordance with the principles of the Declaration of Helsinki. Ethical approval was obtained from the Haliç University Non-Interventional Clinical Research Ethics Committee (decision no. 135; 24 June 2021). All participants were informed about the study procedures and provided written informed consent prior to participation. The reporting of this study conforms to the Strengthening the Reporting of Observational Studies in Epidemiology (STROBE) statement (2007) [15].

### Participants and study size

Participants were recruited from the study villages using snowball sampling. Recruitment began with a small number of initial contacts (seeds) identified by local community members in each village; these seeds then referred other eligible women, and recruitment continued until the target group sizes were reached. The 40-65-year age range was chosen because it spans the menopausal transition and the later reproductive years, when both cumulative occupational musculoskeletal exposure and the risk of urinary incontinence increase. Eligible participants were women aged 40–65 years who were able to complete the questionnaires in Turkish and volunteered to participate. The farmer group comprised women who reported agricultural work as their primary daily occupation and were actively engaged in field work during the production season; the comparison group comprised homemakers from the same rural districts who reported no regular involvement in agricultural activities. Because the questionnaires did not quantify exposure intensity (for example, working hours per week or years of farming), occupational status was defined categorically; the resulting potential for exposure misclassification and for the selection bias inherent in snowball sampling is considered in the Limitations.

Exclusion criteria included neurological or oncological disease, previously diagnosed urogenital disease or history of orthopedic surgery, spinal cord injury, diabetes mellitus, pregnancy, gynecological surgery history, diagnosed psychiatric disorder, or current treatment for urinary incontinence.

The final sample comprised 35 farmers and 35 homemakers, a size constrained by the rural, community-based recruitment of this thesis-based study. At the design stage, the most directly comparable effect available was an odds ratio of 1.81 for the association between occupational status and urinary incontinence [7]. To contextualise statistical power, we conducted a sensitivity power analysis for the comparison of two independent proportions (two-sided α = 0.05) using the statsmodels package; the same analysis is implemented in dedicated power-analysis software such as G*Power 3.1 [16]. With 35 participants per group, the study had 80% power to detect only large between-group differences (Cohen’s h ≈ 0.67, corresponding to roughly a 30-percentage-point difference, or an odds ratio of about 4 at a 30% baseline). Detecting an effect of the magnitude reported for occupational status and urinary incontinence (odds ratio ≈ 1.81) would have required approximately 195 participants per group, so the achieved power for that effect was low (≈ 0.20). The study should therefore be regarded as exploratory and is likely underpowered, particularly for the secondary musculoskeletal and quality-of-life outcomes; non-significant findings are interpreted as inconclusive rather than as evidence of no effect.

### Data collection and instruments

All data were collected face to face by the first author, a physiotherapist, using a structured demographic and clinical form together with the validated Turkish versions of the study questionnaires. To standardise administration, the same examiner conducted all interviews, read each item aloud using fixed wording, and recorded responses directly, which reduced inter-interviewer variability and missing items.

Demographic and clinical variables included age, height, weight, body mass index (BMI, calculated as weight in kilograms divided by height in metres squared and analysed as a continuous variable), menopausal status, marital status, parity-related variables, smoking status, daily water intake, chronic disease, regular medication use, and farmer-specific occupational activities.

Musculoskeletal discomfort was assessed using the Cornell Musculoskeletal Discomfort Questionnaire (CMDQ), which evaluates discomfort across 18 body regions [17]; the validated Turkish version was used (Erdinç et al., 2011) [18]. For each region, participants rated the frequency of discomfort (from never to several times a day) and, on three-point scales, its perceived severity and the degree to which it interfered with their ability to work. Following the standard CMDQ scoring [17, 18], a regional discomfort score was computed as the product of these three ratings (frequency × severity × interference), giving a possible range of 0 to 90 per region, with higher scores indicating greater discomfort; the 18 regional scores were summed to obtain the CMDQ total score (possible range 0 to 1620). For prevalence analyses, a region with a score greater than zero was classified as having discomfort.

Urinary incontinence symptoms were assessed using the International Consultation on Incontinence Questionnaire-Short Form (ICIQ-SF) [19]; the validated Turkish version was used (Çetinel et al., 2004) [20]. The questionnaire evaluates the frequency and amount of urinary leakage, its impact on daily life, and the circumstances under which leakage occurs. The total score ranges from 0 to 21, with higher scores indicating greater symptom burden.

Health-related quality of life was evaluated using the 36-item Short Form Health Survey (SF-36) [21]; the validated Turkish version was used (Koçyiğit et al., 1999) [22], which is based on the original (version 1) SF-36. The SF-36 assesses quality of life across eight domains scored from 0 to 100, where higher scores indicate better perceived health. The eight domain scores were analysed separately; aggregate physical and mental component summary scores (PCS/MCS) were not computed.

### Statistical analysis

Continuous variables were summarised as mean±standard deviation (SD) and, where relevant, median with interquartile range (IQR); categorical variables were summarised as n (%). Normality was examined using distributional inspection and the Shapiro-Wilk test. Between-group comparisons used Mann-Whitney U tests for continuous or ordinal variables and Fisher exact tests for categorical variables.

Urinary incontinence (ICIQ-SF) was treated as the primary outcome, in line with the effect estimate used to plan the study; regional and total musculoskeletal discomfort (CMDQ) and health-related quality of life (SF-36) were treated as secondary outcomes. For region-specific CMDQ outcomes, logistic regression estimated the association between farmer status and the presence of discomfort, adjusting for age, BMI, and menopausal status. For CMDQ total score, ICIQ-SF total score, and SF-36 domains, linear regression models adjusted for age, BMI, menopausal status, and chronic disease.

Spearman correlations were calculated between CMDQ regional scores and SF-36 domains separately within farmers and homemakers. P-values were adjusted using the Benjamini-Hochberg false-discovery-rate procedure [23], with statistical significance set at α = 0.05 (and q < 0.05 for FDR-adjusted comparisons). Analyses used available cases; missing data were limited and arose mainly from incomplete responses across all 18 CMDQ regions, which reduced the sample for the CMDQ total score to 68 (see Results). Analyses were performed in Python 3.11 using the pandas, NumPy, SciPy, and statsmodels packages.

## Results

### Participants and baseline characteristics

A total of 70 women were included in the analysis: 35 farmers and 35 homemakers. The groups were similar in age, height, body weight, and daily water intake. Mean BMI calculated from height and weight was 29.7 ± 4.9 kg/m2 in farmers and 28.0 ± 4.8 kg/m2 in homemakers (*p* = 0.159). Postmenopausal status and chronic disease were more frequent among farmers, whereas the remaining baseline characteristics were generally similar between groups (Table 1).

**Table 1:** Demographic and clinical characteristics

Variable	Farmers (*n* = 35)	Homemakers (*n* = 35)	*p*-value
Age, years	53.7 ± 6.3	51.6 ± 7.5	0.232
Height, cm	161.1 ± 5.8	161.9 ± 4.3	0.307
Weight, kg	76.8 ± 11.8	73.5 ± 13.4	0.196
BMI, kg/m²	29.7 ± 4.9	28.0 ± 4.8	0.159
Water intake, L/day	3.3 ± 0.9	3.0 ± 1.1	0.188
Postmenopausal	27 (77.1)	19 (54.3)	0.077
Chronic disease	15 (42.9)	8 (22.9)	0.126
Regular medication	12 (34.3)	11 (31.4)	1.000
Married	29 (82.9)	27 (77.1)	0.766
Any musculoskeletal discomfort	31 (88.6)	29 (82.9)	0.734

Data are mean ± SD or n (%). BMI was calculated from weight and height.*p*-values are from Mann-Whitney U tests for continuous variables and Fisher exact tests for categorical variables

### Regional musculoskeletal discomfort

**Fig. 1:**
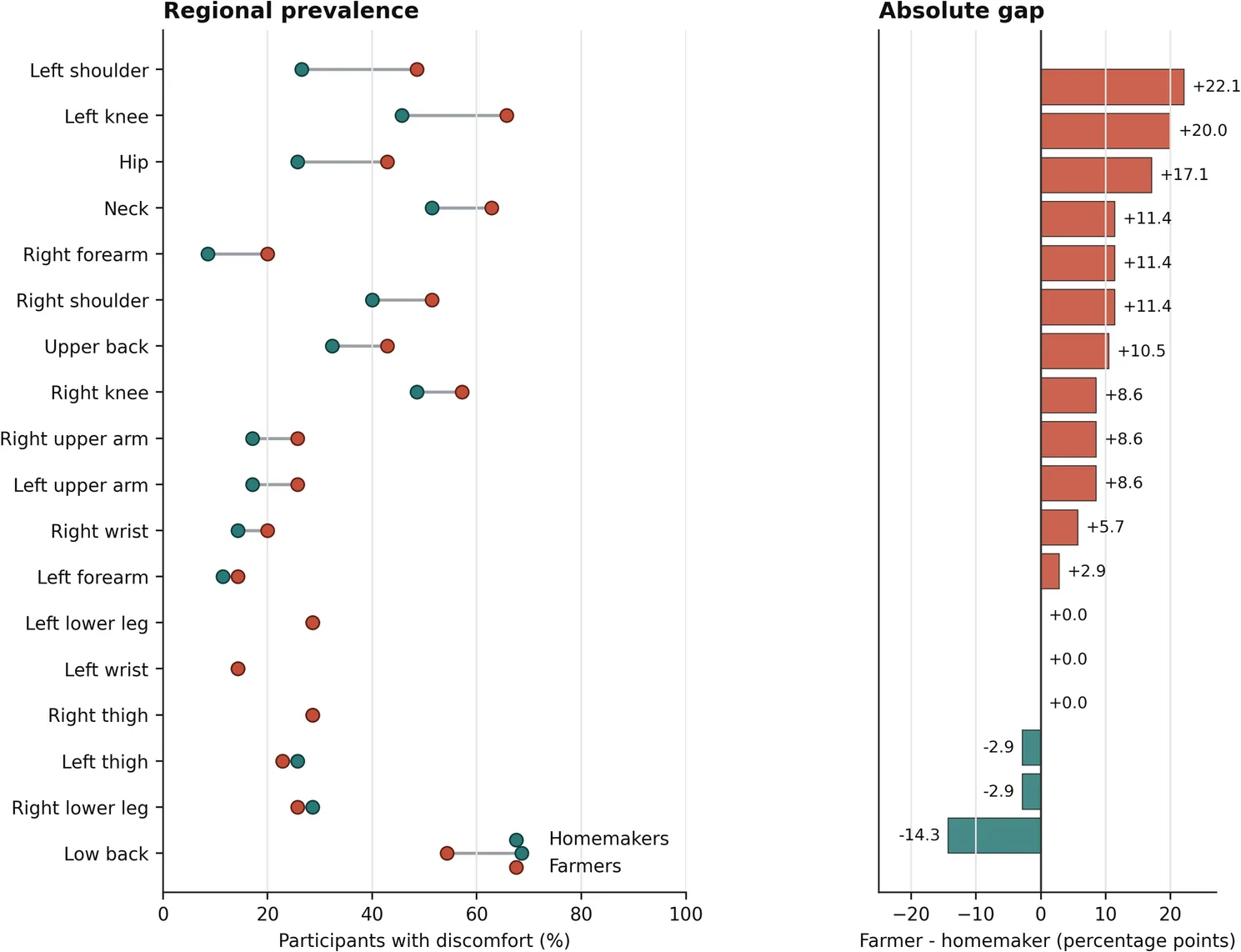
CMDQ discomfort by body region. Red markers denote farmers and green markers denote homemakers. The left panel shows prevalence in each group; the right panel shows farmer-minus-homemaker percentage-point differences. All values are based on regional CMDQ discomfort coded as present versus absent

Farmers had a higher discomfort prevalence in 12 of 18 CMDQ regions, equal prevalence in three regions, and lower prevalence in three regions. The largest unadjusted prevalence differences were observed for the left shoulder (48.6% vs. 26.5%; +22.1% points), left knee (65.7% vs. 45.7%; +20.0), hip (42.9% vs. 25.7%; +17.1), and right forearm (20.0% vs. 8.6%; +11.4) (Fig. 1). No region remained statistically significant after false-discovery-rate correction (minimum q = 0.626; Table 2).

**Table 2:** CMDQ regional discomfort results for the 10 largest positive effect sizes

Body region	Farmers median (IQR)	Homemakers median (IQR)	Prev. F/H (%)	Cohen d (95% CI)	*p*	q
Left knee	20 (0–40)	0 (0–20)	65.7/45.7	0.51 (0.03 to 0.98)	0.042	0.626
Right forearm	0 (0–0)	0 (0–0)	20.0/8.6	0.41 (-0.07 to 0.88)	0.156	0.626
Neck	3 (0–15)	2 (0–6)	62.9/51.4	0.40 (-0.07 to 0.88)	0.143	0.626
Right knee	10 (0–40)	0 (0–30)	57.1/48.6	0.27 (-0.20 to 0.74)	0.348	0.631
Right thigh	0 (0–14)	0 (0–4)	28.6/28.6	0.26 (-0.21 to 0.73)	0.691	0.888
Right upper arm	0 (0–2)	0 (0–0)	25.7/17.1	0.25 (-0.22 to 0.72)	0.351	0.631
Right wrist	0 (0–0)	0 (0–0)	20.0/14.3	0.21 (-0.26 to 0.68)	0.497	0.813
Right shoulder	2 (0–20)	0 (0–8)	51.4/40.0	0.21 (-0.26 to 0.68)	0.276	0.631
Left upper arm	0 (0–4)	0 (0–0)	25.7/17.1	0.20 (-0.27 to 0.67)	0.346	0.631
Left lower leg	0 (0–3)	0 (0–3)	28.6/28.6	0.20 (-0.27 to 0.67)	0.819	0.956

*CMDQ* Cornell Musculoskeletal Discomfort Questionnaire, *F* farmers, *H* homemakers, *q* Benjamini-Hochberg FDR-adjusted p-value. Full 18-region results are provided in Supplementary Table S1

In adjusted logistic models controlling for age, BMI, and menopausal status, farmer status was not independently associated with discomfort in any body region (adjusted p values ranged from 0.081 to 0.993). The largest adjusted odds ratios were observed for the left shoulder, right forearm, hip, and left knee, but confidence intervals were wide and crossed unity (Table 3).

**Table 3:** Selected adjusted logistic models for presence of CMDQ discomfort

Body region	*n*	Prev. F/H (%)	Crude OR	Adjusted OR (95% CI)	*p*
Left shoulder	69	48.6/26.5	2.62	2.12 (0.73–6.13)	0.167
Right forearm	70	20.0/8.6	2.67	2.02 (0.45–9.04)	0.356
Hip	70	42.9/25.7	2.17	1.99 (0.69–5.73)	0.203
Left knee	70	65.7/45.7	2.28	1.74 (0.60–5.08)	0.312
Neck	70	62.9/51.4	1.60	1.40 (0.51–3.81)	0.511
Right shoulder	70	51.4/40.0	1.59	1.36 (0.50–3.68)	0.551
Low back	70	54.3/68.6	0.54	0.37 (0.12–1.13)	0.081

Adjusted models included farmer status, age, BMI, and menopausal status. Full results for all 18 regions are provided in Supplementary Table S2

### Urinary incontinence

ICIQ-SF total score tended to be higher in farmers than homemakers (4.0 ± 3.8 vs. 3.0 ± 4.2), but the difference was not statistically significant (*p* = 0.127). After adjustment for age, BMI, menopausal status, and chronic disease, the estimated farmer-homemaker difference was 0.56 points (95% CI -1.45 to 2.58; *p* = 0.578).

Trigger-context responses showed nonsignificant tendencies toward more leakage before reaching the toilet (51.4% vs. 28.6%; *p* = 0.087) and more leakage during physical activity/exercise (31.4% vs. 11.4%; *p* = 0.078) among farmers.

### Health-related quality of life

SF-36 scores were numerically lower among farmers across all eight domains. The largest unadjusted difference was observed for role emotional (27.6 ± 34.8 vs. 40.0 ± 37.8; *p* = 0.122). After adjustment for age, BMI, menopausal status, and chronic disease, no SF-36 domain differed significantly between groups (adjusted p values ranged from 0.296 to 0.886; Table 4).

**Table 4:** CMDQ total, ICIQ-SF total, and SF-36 outcomes

Outcome	*n*	Farmers mean ± SD	Homemakers mean ± SD	Unadj. *p*	Adjusted β (95% CI)	Adj. *p*
CMDQ total score	68	191.2 ± 223.4	136.3 ± 153.5	0.250	0.79 (-92.44 to 94.01)	0.987
ICIQ-SF total score	70	4.0 ± 3.8	3.0 ± 4.2	0.127	0.56 (-1.45 to 2.58)	0.578
SF-36 Physical functioning	70	62.4 ± 21.4	68.4 ± 24.4	0.289	0.74 (-9.55 to 11.03)	0.886
SF-36 Role physical	70	42.9 ± 40.9	45.7 ± 47.2	0.800	4.95 (-16.65 to 26.55)	0.649
SF-36 Role emotional	70	27.6 ± 34.8	40.0 ± 37.8	0.122	-9.78 (-28.34 to 8.78)	0.296
SF-36 Vitality	70	46.0 ± 20.1	46.5 ± 22.2	0.939	3.72 (-6.61 to 14.05)	0.474
SF-36 Mental health	70	56.3 ± 19.1	59.1 ± 21.0	0.596	1.33 (-8.39 to 11.06)	0.785
SF-36 Social functioning	70	77.9 ± 20.4	80.2 ± 22.6	0.490	-2.17 (-13.09 to 8.74)	0.692
SF-36 Bodily pain	70	52.4 ± 19.2	54.9 ± 25.7	0.624	2.07 (-8.72 to 12.85)	0.703
SF-36 General health	70	54.9 ± 15.1	55.4 ± 17.1	0.786	3.80 (-3.76 to 11.35)	0.319

Linear models adjusted for age, BMI, menopausal status, and chronic disease. The study sample was *n* = 70; the CMDQ total score row includes *n* = 68 because complete responses across all 18 CMDQ regions were required. Higher SF-36 scores indicate better health-related quality of life

### Correlations between regional discomfort and quality of life

**Fig. 2:**
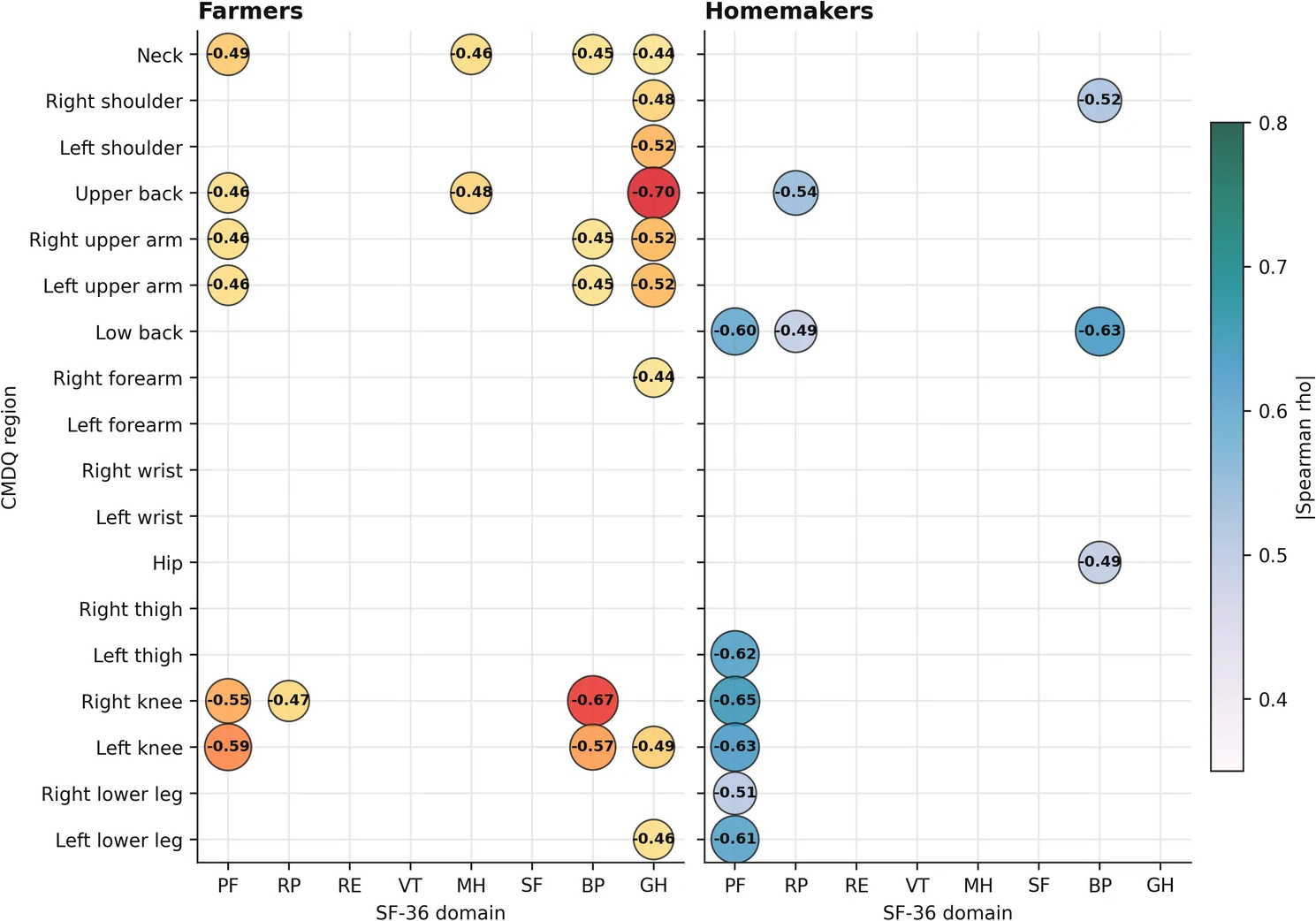
CMDQ-SF-36 correlation map. Only FDR-significant inverse Spearman correlations are shown. Bubble size and colour intensity represent stronger absolute correlations; the number inside each bubble is rho. CMDQ, Cornell Musculoskeletal Discomfort Questionnaire; FDR, false discovery rate; SF-36, 36-item Short Form Health Survey

Several CMDQ-SF-36 correlations remained significant after false discovery rate (FDR) correction. Among farmers, greater upper-back discomfort was correlated with lower general health scores (rho=-0.700, *p* < 0.001, q < 0.001), and greater right-knee discomfort with lower bodily pain scores (rho=-0.672, *p* < 0.001, q < 0.001). Among homemakers, greater low-back discomfort was correlated with lower physical functioning (rho=-0.596, *p* < 0.001, q = 0.004) and bodily pain scores (rho=-0.632, *p* < 0.001, q = 0.003) (Fig. 2).

## Discussion

This cross-sectional comparative study found that female farmers aged 40–65 years had a more widespread regional distribution of musculoskeletal discomfort than homemakers from the same rural districts. Farmers showed higher discomfort prevalence in most CMDQ regions, especially the left shoulder, left knee, hip, and right forearm. However, adjusted region-specific models and FDR-corrected tests did not provide strong evidence of independent occupational-group effects. Therefore, the findings should be interpreted as exploratory rather than as evidence of causal occupational effects.

The observed regional pattern is consistent with the physical demands of agricultural work. Activities such as harvesting, planting, milking, carrying, and field work may involve repetitive upper-extremity movements together with prolonged standing, squatting, kneeling, and trunk flexion. Previous studies have similarly reported a high burden of musculoskeletal symptoms among farmers and farm workers [1–5], and a recent systematic review and meta-analysis of agricultural workers in low- and middle-income countries confirmed that musculoskeletal pain—most often low back pain—is highly prevalent and is associated with heavy lifting and repetitive movements [24]. The present findings extend this literature by highlighting the anatomical regions in which discomfort appeared most prominent in this rural female population.

Unexpectedly, low back discomfort was more prevalent among homemakers than farmers, despite the general expectation that farming increases spinal load. This observation may indicate that domestic activities performed in rural settings also impose substantial physical demands on the lumbar region. Accordingly, future studies should measure domestic and agricultural exposures together rather than treating homemakers as unexposed.

Findings related to urinary incontinence should be interpreted cautiously. Farmers demonstrated a tendency toward higher ICIQ-SF scores and more frequent self-reported leakage during physical activity/exercise and before reaching the toilet, but these differences were not statistically significant. These patterns may reflect two partially overlapping mechanisms. The first is exertion-related leakage during tasks that increase intra-abdominal pressure, such as lifting, carrying, and sustained physical work; recent meta-analytic evidence similarly links high-impact, high-intra-abdominal-pressure activity to a high prevalence of predominantly stress-type urinary incontinence in women [25]. The second is urgency-related leakage, potentially associated with delayed toileting or limited toilet access during field work. The cross-sectional design and modest sample do not allow attribution of urinary symptoms to farming; nonetheless, the coexistence of musculoskeletal discomfort and urinary leakage is clinically relevant, because both may restrict participation and perceived quality of life [7–14].

SF-36 scores tended to be lower among farmers; however, adjusted between-group differences were not statistically significant. The correlation analyses were informative because they showed that regional discomfort, rather than occupational group alone, was associated with quality-of-life domains. These findings highlight the importance of body-region-specific assessment in rural women’s health research.

The clinical implications are practical but should not be overstated. In occupational and women’s-health physiotherapy, asking female farmers about knee, shoulder, hip, and upper-back discomfort may help identify individuals who would benefit from targeted interventions. These could include ergonomic and task-modification advice (for example, optimising load handling, alternating between sustained postures, and incorporating rest breaks during field work), region-specific strengthening and flexibility exercises, and onward referral when symptoms are disabling. Because farmers also tended to report more activity-related and urgency-related leakage, enquiry about urinary symptoms and toilet access during work may identify women who would benefit from pelvic-floor muscle training, bladder and fluid-management education, and conservative physiotherapy, which are first-line, low-risk options for stress and mixed urinary incontinence. Integrating brief musculoskeletal and pelvic-floor screening into rural primary-care and occupational-health contacts may therefore be a pragmatic way to reach this underserved group, although the present exploratory findings cannot establish that such screening improves outcomes.

This study has several limitations. The principal limitation was the limited assessment of occupational and domestic exposure. Because exposure was defined categorically by occupational group and self-reported activities, rather than by daily working hours, weekly frequency, years of agricultural or domestic work, seasonal workload, task duration, load weight, postural exposure, rest breaks, or toilet access, exposure misclassification is likely—particularly because rural homemakers may also perform physically demanding domestic tasks, which would bias group comparisons toward the null. Second, the cross-sectional design precludes causal inference. Third, the sample was modest and recruited by snowball sampling from a single Turkish province, which limits generalisability and precision and, as the sensitivity analysis showed, left the study underpowered for the secondary musculoskeletal and quality-of-life outcomes; the predominantly non-significant adjusted results may therefore reflect type II error rather than a true absence of effect. Fourth, detailed ergonomic assessment, pelvic-floor muscle strength, obstetric history, and objective physical-performance measures were not available, and some questionnaire items had missing values. Finally, multiple testing increased the risk of chance findings, although this was mitigated by FDR correction. These limitations reinforce the exploratory, hypothesis-generating nature of the study.

## Conclusions

In this exploratory study, female farmers showed a broader distribution of musculoskeletal discomfort than rural homemakers, but adjusted and multiple-testing-corrected analyses did not confirm independent occupational-group effects, and the urinary incontinence and quality-of-life differences were small and non-significant. The findings are therefore hypothesis-generating rather than confirmatory. They nonetheless support consideration of combined musculoskeletal and pelvic-floor assessment in rural women’s health, and future studies should use detailed agricultural and domestic exposure metrics while evaluating body-region discomfort, urinary symptoms, and quality of life together in adequately powered samples.

## Supplementary Information

Supplementary Material 1.

## Data Availability

The datasets analysed during the current study are available from the corresponding author on reasonable request.

## Abbreviations

BMI: Body mass index
CI: Confidence interval
CMDQ: Cornell Musculoskeletal Discomfort Questionnaire
FDR: False discovery rate
ICIQ-SF: International Consultation on Incontinence Questionnaire-Short Form
OR: Odds ratio
SF-36: 36-Item Short Form Health Survey
STROBE: Strengthening the Reporting of Observational Studies in Epidemiology

## References

[1] Walker-Bone K, Palmer KT. Musculoskeletal disorders in farmers and farm workers. Occup Med (Lond). 2002;52(8):441–50. 10.1093/occmed/52.8.441. 12488514

[2] Osborne A, Blake C, Fullen BM, Meredith D, Phelan J, McNamara J, et al. Prevalence of musculoskeletal disorders among farmers: a systematic review. Am J Ind Med. 2012;55(2):143–58. 10.1002/ajim.21033. 22069159

[3] Barneo-Alcántara M, Díaz-Pérez M, Gómez-Galán M, Carreño-Ortega Á, Callejón-Ferre ÁJ. Musculoskeletal disorders in agriculture: a review from Web of Science Core Collection. Agronomy. 2021;11(10):2017. 10.3390/agronomy11102017.

[4] Dianat I, Afshari D, Sarmasti N, Sangdeh MS, Azaddel R. Work posture, working conditions and musculoskeletal outcomes in agricultural workers. Int J Ind Ergon. 2020;77:102941. 10.1016/j.ergon.2020.102941.

[5] Shin DS, Jeong BY. Older female farmers and modeling of occupational hazards, wellbeing, and sleep-related problems on musculoskeletal pains. Int J Environ Res Public Health. 2022;19(12):7274. 10.3390/ijerph19127274. PMC9223070 35742520

[6] Kadota JL, McCoy SI, Bates MN, Mnyippembe A, Njau PF, Prata N, et al. The impact of heavy load carrying on musculoskeletal pain and disability among women in Shinyanga Region, Tanzania. Ann Glob Health. 2020;86(1):17. 10.5334/aogh.2470. PMC7034319 32110509

[7] Kim Y, Kwak Y. Urinary incontinence in women in relation to occupational status. Women Health. 2017;57(1):1–18. 10.1080/03630242.2016.1150387. 26853676

[8] Åström Y, Asklund I, Lindam A, Sjöström M. Quality of life in women with urinary incontinence seeking care using e-health. BMC Womens Health. 2021;21:337. 10.1186/s12905-021-01477-0. PMC8454026 34544393

[9] Zhang RQ, Xia MC, Cui F, Chen JW, Bian XD, Xie HJ. Epidemiological survey of adult female stress urinary incontinence. BMC Womens Health. 2021;21:172. 10.1186/s12905-021-01319-z. PMC8061196 33888113

[10] Zhu Y, Wei J, Yang X, Zhu W, Zhang W. Investigation on prevalence and risk factors associated with genitourinary syndrome of menopause in middle-aged and older women in Beijing community: a cross sectional study. BMC Womens Health. 2022;22:558. 10.1186/s12905-022-02099-w. PMC9801667 36585649

[11] Long T, Cheng B, Zhang K. Abdominal obesity as assessed by anthropometric measures associates with urinary incontinence in females: findings from NHANES 2005–2018. BMC Womens Health. 2024;24:212. 10.1186/s12905-024-03059-2. PMC10986057 38566030

[12] Sawettikamporn W, Sarit-apirak S, Manonai J. Attitudes and barriers to pelvic floor muscle exercises of women with stress urinary incontinence. BMC Womens Health. 2022;22:477. 10.1186/s12905-022-02067-4. PMC9701389 36435776

[13] Neumann PB, Grimmer KA, Deenadayalan Y. Pelvic floor muscle training and adjunctive therapies for the treatment of stress urinary incontinence in women: a systematic review. BMC Womens Health. 2006;6:11. 10.1186/1472-6874-6-11. PMC1586224 16805910

[14] Pizzol D, Demurtas J, Celotto S, Maggi S, Smith L, Angiolelli G, et al. Urinary incontinence and quality of life: a systematic review and meta-analysis. Aging Clin Exp Res. 2021;33(1):25–35. 10.1007/s40520-020-01712-y. PMC7897623 32964401

[15] von Elm E, Altman DG, Egger M, Pocock SJ, Gøtzsche PC, Vandenbroucke JP, et al. The Strengthening the Reporting of Observational Studies in Epidemiology (STROBE) statement: guidelines for reporting observational studies. PLoS Med. 2007;4(10):e296. 10.1371/journal.pmed.0040296. PMC2020495 17941714

[16] Faul F, Erdfelder E, Lang AG, Buchner A. G*Power 3: a flexible statistical power analysis program for the social, behavioral, and biomedical sciences. Behav Res Methods. 2007;39(2):175–91. 10.3758/BF03193146. 17695343

[17] Hedge A, Cornell Human Factors and Ergonomics Research Group. Cornell Musculoskeletal Discomfort Questionnaires (CMDQ). Cornell University Ergonomics Web. Accessed 15 Jul 2021. Available from: https://ergo.human.cornell.edu/ahmsquest.html.

[18] Erdinc O, Hot K, Ozkaya M. Turkish version of the Cornell Musculoskeletal Discomfort Questionnaire: cross-cultural adaptation and validation. Work. 2011;39(3):251–60. 10.3233/WOR-2011-1173. 21709361

[19] Avery K, Donovan J, Peters TJ, Shaw C, Gotoh M, Abrams P. ICIQ: a brief and robust measure for evaluating the symptoms and impact of urinary incontinence. Neurourol Urodyn. 2004;23(4):322–30. 10.1002/nau.20041. 15227649

[20] Çetinel B, Özkan B, Can G. [Validation study of the Turkish version of the ICIQ-SF]. Türk Üroloji Dergisi. (Turkish J Urology). 2004;30(3):332–8. Turkish.

[21] Ware JE Jr, Sherbourne CD. The MOS 36-item short-form health survey (SF-36): I. Conceptual framework and item selection. Med Care. 1992;30(6):473–83. 10.1097/00005650-199206000-00002. 1593914

[22] Koçyiğit H, Aydemir Ö, Fişek G, Ölmez N, Memiş A. [Reliability and validity of the Turkish version of the Short Form-36 (SF-36)]. İlaç ve Tedavi Dergisi. (Journal Drugs Therapy). 1999;12:102–6. Turkish.

[23] Benjamini Y, Hochberg Y. Controlling the false discovery rate: a practical and powerful approach to multiple testing. J R Stat Soc Ser B Stat Methodol. 1995;57(1):289–300. 10.1111/j.2517-6161.1995.tb02031.x.

[24] Shivakumar M, Welsh V, Bajpai R, Helliwell T, Mallen C, Robinson M, et al. Musculoskeletal disorders and pain in agricultural workers in low- and middle-income countries: a systematic review and meta-analysis. Rheumatol Int. 2024;44(2):235–47. 10.1007/s00296-023-05500-5. PMC10796632 37999798

[25] Dominguez-Antuña E, Diz JC, Suárez-Iglesias D, Ayán C. Prevalence of urinary incontinence in female CrossFit athletes: a systematic review with meta-analysis. Int Urogynecol J. 2023;34(3):621–34. 10.1007/s00192-022-05244-z. PMC9150382 35635565

