# Supplementary material for: Musculoskeletal discomfort, urinary incontinence, and health-related quality of life in female farmers aged 40–65 years: a cross-sectional comparative study from rural Türkiye
Source: BMC Womens Health. 2026 Jul 17;26:516. doi: 10.1186/s12905-026-04682-x (PMC13660044; doi:10.1186/s12905-026-04682-x)
Supplement: Supplementary file 1 [file 12905_2026_4682_MOESM1_ESM.docx]

Supplementary Material

**Musculoskeletal Discomfort, Urinary Incontinence, and Health-Related Quality of Life in Female Farmers Aged 40-65 Years: A Cross-Sectional Comparative Study from Rural Türkiye**

This supplementary file provides full regional CMDQ analyses, complete adjusted model results, urinary leakage trigger-context data, correlation analyses, and a STROBE checklist.

# Supplementary Table S1. Complete 18-region CMDQ results

| **Body region** | **n F/H** | **Farmers median (IQR)** | **Homemakers median (IQR)** | **Mean±SD F** | **Mean±SD H** | **Prev. F** | **Prev. H** | **Diff.** | **Cohen d (95% CI)** | **p** | **q** |
| --- | --- | --- | --- | --- | --- | --- | --- | --- | --- | --- | --- |
| Left knee | 35/35 | 20 (0-40) | 0 (0-20) | 26.2 ± 31.0 | 12.9 ± 20.6 | 65.7 | 45.7 | +20.0 | 0.51 (0.03 to 0.98) | **0.042** | 0.626 |
| Right forearm | 35/35 | 0 (0-0) | 0 (0-0) | 4.4 ± 10.8 | 1.1 ± 4.1 | 20.0 | 8.6 | +11.4 | 0.41 (-0.07 to 0.88) | 0.156 | 0.626 |
| Neck | 35/35 | 3 (0-15) | 2 (0-6) | 15.6 ± 26.4 | 6.8 ± 16.1 | 62.9 | 51.4 | +11.4 | 0.40 (-0.07 to 0.88) | 0.143 | 0.626 |
| Right knee | 35/35 | 10 (0-40) | 0 (0-30) | 26.3 ± 33.1 | 18.2 ± 25.9 | 57.1 | 48.6 | +8.6 | 0.27 (-0.20 to 0.74) | 0.348 | 0.631 |
| Right thigh | 35/35 | 0 (0-14) | 0 (0-4) | 9.4 ± 18.8 | 5.3 ± 12.1 | 28.6 | 28.6 | +0.0 | 0.26 (-0.21 to 0.73) | 0.691 | 0.888 |
| Right upper arm | 35/35 | 0 (0-2) | 0 (0-0) | 5.1 ± 9.9 | 2.9 ± 7.6 | 25.7 | 17.1 | +8.6 | 0.25 (-0.22 to 0.72) | 0.351 | 0.631 |
| Right wrist | 35/35 | 0 (0-0) | 0 (0-0) | 4.1 ± 10.3 | 2.3 ± 6.8 | 20.0 | 14.3 | +5.7 | 0.21 (-0.26 to 0.68) | 0.497 | 0.813 |
| Right shoulder | 35/35 | 2 (0-20) | 0 (0-8) | 14.4 ± 26.1 | 9.5 ± 21.8 | 51.4 | 40.0 | +11.4 | 0.21 (-0.26 to 0.68) | 0.276 | 0.631 |
| Left upper arm | 35/35 | 0 (0-4) | 0 (0-0) | 5.2 ± 9.9 | 3.2 ± 9.2 | 25.7 | 17.1 | +8.6 | 0.20 (-0.27 to 0.67) | 0.346 | 0.631 |
| Left lower leg | 35/35 | 0 (0-3) | 0 (0-3) | 9.8 ± 21.3 | 6.0 ± 16.2 | 28.6 | 28.6 | +0.0 | 0.20 (-0.27 to 0.67) | 0.819 | 0.956 |
| Left forearm | 35/35 | 0 (0-0) | 0 (0-0) | 4.1 ± 10.8 | 2.4 ± 8.5 | 14.3 | 11.4 | +2.9 | 0.17 (-0.29 to 0.64) | 0.671 | 0.888 |
| Left thigh | 35/35 | 0 (0-0) | 0 (0-2) | 8.0 ± 18.5 | 5.6 ± 16.0 | 22.9 | 25.7 | -2.9 | 0.14 (-0.33 to 0.61) | 0.956 | 0.956 |
| Right lower leg | 35/35 | 0 (0-2) | 0 (0-3) | 8.4 ± 20.6 | 5.9 ± 14.6 | 25.7 | 28.6 | -2.9 | 0.14 (-0.33 to 0.61) | 0.887 | 0.956 |
| Hip | 35/35 | 0 (0-20) | 0 (0-2) | 12.2 ± 20.7 | 9.5 ± 20.8 | 42.9 | 25.7 | +17.1 | 0.13 (-0.34 to 0.60) | 0.209 | 0.626 |
| Left shoulder | 35/34 | 0 (0-14) | 0 (0-1) | 11.2 ± 22.1 | 8.5 ± 22.3 | 48.6 | 26.5 | +22.1 | 0.12 (-0.35 to 0.59) | 0.098 | 0.626 |
| Upper back | 35/34 | 0 (0-7) | 0 (0-7) | 7.7 ± 16.9 | 6.2 ± 10.7 | 42.9 | 32.4 | +10.5 | 0.10 (-0.37 to 0.57) | 0.600 | 0.888 |
| Left wrist | 35/35 | 0 (0-0) | 0 (0-0) | 3.8 ± 10.3 | 3.0 ± 9.5 | 14.3 | 14.3 | +0.0 | 0.08 (-0.39 to 0.55) | 0.954 | 0.956 |
| Low back | 35/35 | 7 (0-20) | 10 (0-42) | 15.4 ± 22.3 | 24.6 ± 29.4 | 54.3 | 68.6 | -14.3 | -0.35 (-0.83 to 0.12) | 0.187 | 0.626 |

*p-values from Mann-Whitney U tests; q-values from Benjamini-Hochberg FDR correction across 18 regions. Rows are sorted by descending Cohen's d.*

# Supplementary Table S2. Full multivariable logistic regression results

| **Body region** | **n** | **Prev. F (%)** | **Prev. H (%)** | **Crude OR** | **Adjusted OR (95% CI)** | **p** |
| --- | --- | --- | --- | --- | --- | --- |
| Left shoulder | 69 | 48.6 | 26.5 | 2.62 | 2.12 (0.73-6.13) | 0.167 |
| Right forearm | 70 | 20.0 | 8.6 | 2.67 | 2.02 (0.45-9.04) | 0.356 |
| Hip | 70 | 42.9 | 25.7 | 2.17 | 1.99 (0.69-5.73) | 0.203 |
| Left knee | 70 | 65.7 | 45.7 | 2.28 | 1.74 (0.60-5.08) | 0.312 |
| Neck | 70 | 62.9 | 51.4 | 1.60 | 1.40 (0.51-3.81) | 0.511 |
| Left upper arm | 70 | 25.7 | 17.1 | 1.67 | 1.36 (0.40-4.67) | 0.623 |
| Right shoulder | 70 | 51.4 | 40.0 | 1.59 | 1.36 (0.50-3.68) | 0.551 |
| Right upper arm | 70 | 25.7 | 17.1 | 1.67 | 1.34 (0.40-4.49) | 0.639 |
| Right wrist | 70 | 20.0 | 14.3 | 1.50 | 1.16 (0.30-4.44) | 0.827 |
| Upper back | 69 | 42.9 | 32.4 | 1.57 | 1.12 (0.39-3.23) | 0.833 |
| Right knee | 70 | 57.1 | 48.6 | 1.41 | 1.00 (0.35-2.81) | 0.993 |
| Right thigh | 70 | 28.6 | 28.6 | 1.00 | 0.96 (0.33-2.84) | 0.943 |
| Left forearm | 70 | 14.3 | 11.4 | 1.29 | 0.94 (0.22-4.10) | 0.937 |
| Left wrist | 70 | 14.3 | 14.3 | 1.00 | 0.73 (0.18-2.99) | 0.664 |
| Left thigh | 70 | 22.9 | 25.7 | 0.86 | 0.66 (0.21-2.13) | 0.491 |
| Left lower leg | 70 | 28.6 | 28.6 | 1.00 | 0.63 (0.20-1.98) | 0.425 |
| Right lower leg | 70 | 25.7 | 28.6 | 0.87 | 0.58 (0.18-1.84) | 0.354 |
| Low back | 70 | 54.3 | 68.6 | 0.54 | 0.37 (0.12-1.13) | 0.081 |

*Outcome: presence of CMDQ discomfort (score >0) in each body region. Adjusted models included farmer status, age, BMI, and menopausal status. Reference group: homemakers.*

# Supplementary Table S3. ICIQ-SF trigger-context prevalences

| **Trigger-context item** | **Farmers, n (%)** | **Homemakers, n (%)** | **Difference (pp)** | **p** |
| --- | --- | --- | --- | --- |
| Never | 14 (40.0) | 21 (60.0) | -20.0 | 0.151 |
| Leakage before reaching the toilet | 18 (51.4) | 10 (28.6) | +22.9 | 0.087 |
| Leakage when coughing or sneezing | 17 (48.6) | 13 (37.1) | +11.4 | 0.469 |
| Leakage during sleep | 3 (8.6) | 0 (0.0) | +8.6 | 0.239 |
| Leakage during physical activity/exercise | 11 (31.4) | 4 (11.4) | +20.0 | 0.078 |
| Leakage after voiding while dressing | 2 (5.7) | 2 (5.7) | +0.0 | 1.000 |
| Leakage without apparent reason | 2 (5.7) | 0 (0.0) | +5.7 | 0.493 |
| Leakage all the time | 0 (0.0) | 0 (0.0) | +0.0 | 1.000 |

*The trigger-context item allows multiple responses. Percentages can therefore sum to more than 100%. These responses were interpreted as leakage contexts, not clinical diagnostic categories.*

# Supplementary Table S4. Full adjusted continuous-outcome models

| **Outcome** | **n** | **Farmers mean±SD** | **Homemakers mean±SD** | **Unadjusted p** | **Cohen d (95% CI)** | **Adjusted β (95% CI)** | **Adjusted p** |
| --- | --- | --- | --- | --- | --- | --- | --- |
| CMDQ total score | 68 | 191.2 ± 223.4 | 136.3 ± 153.5 | 0.250 | 0.28 (-0.19 to 0.76) | 0.79 (-92.44 to 94.01) | 0.987 |
| ICIQ-SF total score | 70 | 4.0 ± 3.8 | 3.0 ± 4.2 | 0.127 | 0.27 (-0.21 to 0.74) | 0.56 (-1.45 to 2.58) | 0.578 |
| SF-36 Physical functioning | 70 | 62.4 ± 21.4 | 68.4 ± 24.4 | 0.289 | -0.26 (-0.73 to 0.21) | 0.74 (-9.55 to 11.03) | 0.886 |
| SF-36 Role physical | 70 | 42.9 ± 40.9 | 45.7 ± 47.2 | 0.800 | -0.06 (-0.53 to 0.40) | 4.95 (-16.65 to 26.55) | 0.649 |
| SF-36 Role emotional | 70 | 27.6 ± 34.8 | 40.0 ± 37.8 | 0.122 | -0.34 (-0.81 to 0.13) | -9.78 (-28.34 to 8.78) | 0.296 |
| SF-36 Vitality | 70 | 46.0 ± 20.1 | 46.5 ± 22.2 | 0.939 | -0.02 (-0.49 to 0.44) | 3.72 (-6.61 to 14.05) | 0.474 |
| SF-36 Mental health | 70 | 56.3 ± 19.1 | 59.1 ± 21.0 | 0.596 | -0.14 (-0.61 to 0.33) | 1.33 (-8.39 to 11.06) | 0.785 |
| SF-36 Social functioning | 70 | 77.9 ± 20.4 | 80.2 ± 22.6 | 0.490 | -0.11 (-0.58 to 0.36) | -2.17 (-13.09 to 8.74) | 0.692 |
| SF-36 Bodily pain | 70 | 52.4 ± 19.2 | 54.9 ± 25.7 | 0.624 | -0.11 (-0.58 to 0.36) | 2.07 (-8.72 to 12.85) | 0.703 |
| SF-36 General health | 70 | 54.9 ± 15.1 | 55.4 ± 17.1 | 0.786 | -0.04 (-0.50 to 0.43) | 3.80 (-3.76 to 11.35) | 0.319 |

Linear models adjusted for age, BMI, menopausal status, and chronic disease. The study sample was n=70; the CMDQ total score row includes n=68 because complete responses across all 18 CMDQ regions were required.

# Supplementary Table S5. FDR-significant CMDQ-SF-36 Spearman correlations

| **Group** | **CMDQ region** | **SF-36 domain** | **n** | **rho** | **p** | **q** |
| --- | --- | --- | --- | --- | --- | --- |
| Farmers | Upper back | General health | 35 | -0.700 | **<0.001** | **<0.001** |
| Farmers | Right knee | Bodily pain | 35 | -0.672 | **<0.001** | **<0.001** |
| Farmers | Left knee | Physical functioning | 35 | -0.592 | **<0.001** | **0.009** |
| Farmers | Left knee | Bodily pain | 35 | -0.570 | **<0.001** | **0.013** |
| Farmers | Right knee | Physical functioning | 35 | -0.546 | **<0.001** | **0.020** |
| Farmers | Left shoulder | General health | 35 | -0.525 | **0.001** | **0.024** |
| Farmers | Right upper arm | General health | 35 | -0.522 | **0.001** | **0.024** |
| Farmers | Left upper arm | General health | 35 | -0.522 | **0.001** | **0.024** |
| Farmers | Neck | Physical functioning | 35 | -0.493 | **0.003** | **0.042** |
| Farmers | Left knee | General health | 35 | -0.488 | **0.003** | **0.042** |
| Farmers | Upper back | Mental health | 35 | -0.478 | **0.004** | **0.044** |
| Farmers | Right shoulder | General health | 35 | -0.478 | **0.004** | **0.044** |
| Farmers | Right knee | Role physical | 35 | -0.473 | **0.004** | **0.046** |
| Farmers | Neck | Mental health | 35 | -0.461 | **0.005** | **0.046** |
| Farmers | Upper back | Physical functioning | 35 | -0.459 | **0.006** | **0.046** |
| Farmers | Right upper arm | Physical functioning | 35 | -0.458 | **0.006** | **0.046** |
| Farmers | Left upper arm | Physical functioning | 35 | -0.458 | **0.006** | **0.046** |
| Farmers | Left lower leg | General health | 35 | -0.457 | **0.006** | **0.046** |
| Farmers | Right upper arm | Bodily pain | 35 | -0.450 | **0.007** | **0.047** |
| Farmers | Left upper arm | Bodily pain | 35 | -0.450 | **0.007** | **0.047** |
| Farmers | Neck | Bodily pain | 35 | -0.449 | **0.007** | **0.047** |
| Farmers | Neck | General health | 35 | -0.444 | **0.008** | 0.050 |
| Farmers | Right forearm | General health | 35 | -0.442 | **0.008** | 0.050 |
| Homemakers | Right knee | Physical functioning | 35 | -0.645 | **<0.001** | **0.003** |
| Homemakers | Low back | Bodily pain | 35 | -0.632 | **<0.001** | **0.003** |
| Homemakers | Left knee | Physical functioning | 35 | -0.628 | **<0.001** | **0.003** |
| Homemakers | Left thigh | Physical functioning | 35 | -0.619 | **<0.001** | **0.003** |
| Homemakers | Left lower leg | Physical functioning | 35 | -0.611 | **<0.001** | **0.003** |
| Homemakers | Low back | Physical functioning | 35 | -0.596 | **<0.001** | **0.004** |
| Homemakers | Upper back | Role physical | 34 | -0.542 | **<0.001** | **0.019** |
| Homemakers | Right shoulder | Bodily pain | 35 | -0.523 | **0.001** | **0.023** |
| Homemakers | Right lower leg | Physical functioning | 35 | -0.505 | **0.002** | **0.031** |
| Homemakers | Hip | Bodily pain | 35 | -0.494 | **0.003** | **0.036** |
| Homemakers | Low back | Role physical | 35 | -0.491 | **0.003** | **0.036** |

*Only correlations with q<0.05 are shown. q-values were calculated within each occupational group across the 18 CMDQ regions × 8 SF-36 domains.*

# Supplementary Table S6. STROBE checklist for cross-sectional studies

| **Section/topic** | **Item** | **Recommendation** | **Location** |
| --- | --- | --- | --- |
| Title and abstract | 1 | Study design indicated in title and abstract | Title; Abstract |
| Background/rationale | 2 | Scientific background and rationale explained | Introduction |
| Objectives | 3 | Objectives and exploratory hypotheses stated | Introduction |
| Study design | 4 | Key elements of cross-sectional design presented | Methods 2.1 |
| Setting | 5 | Location, dates, and setting described | Methods 2.1 |
| Participants | 6 | Eligibility criteria and recruitment described | Methods 2.2 |
| Variables | 7 | Outcomes, exposure, and covariates defined | Methods 2.3-2.4 |
| Data sources/measurement | 8 | Questionnaires and validation references reported | Methods 2.3 |
| Bias | 9 | Sampling and measurement limitations discussed | Discussion |
| Study size | 10 | Study-size rationale described | Methods 2.2 |
| Quantitative variables | 11 | Variable handling and score construction described | Methods 2.3-2.4 |
| Statistical methods | 12 | Statistical tests, adjustment, FDR, and missing data described | Methods 2.4 |
| Participants | 13 | Participant numbers reported | Results 3.1 |
| Descriptive data | 14 | Baseline characteristics reported | Table 1 |
| Outcome data | 15 | CMDQ, ICIQ-SF, and SF-36 outcomes reported | Tables 2-4; Supplement |
| Main results | 16 | Unadjusted and adjusted estimates reported | Tables 2-4; Supplement |
| Other analyses | 17 | Correlation analyses reported | Results 3.5; Table S5 |
| Key results | 18 | Key findings summarised | Discussion |
| Limitations | 19 | Bias, imprecision, and generalisability addressed | Discussion |
| Interpretation | 20 | Cautious interpretation provided | Discussion |
| Generalisability | 21 | Single-province rural sample addressed | Discussion |
| Funding | 22 | Funding statement provided | Declarations |

*Checklist adapted for the manuscript's cross-sectional design.*
